# Micromagnetic insights on in-plane magnetization rotation and propagation of magnetization waves in nanowires

**DOI:** 10.1038/s41598-023-40515-9

**Published:** 2023-08-18

**Authors:** Abir Shadman, Jian-Gang Zhu

**Affiliations:** Electrical and Computer Engineering, Carnegie Mellon University, Pittsburgh, PA 15217 USA

**Keywords:** Electrical and electronic engineering, Ferromagnetism, Magnetic properties and materials, Spintronics

## Abstract

Utilizing micromagnetic modeling, we have explained the unprobed characteristics of 360° full cycle in-plane magnetization rotation and the resulting propagation of a magnetization wave along a ferromagnet nanowire. The magnetization wave, which is generated by setting off spin oscillation at one end of a ferromagnetic strip, propagates till the end of the wire. A perpendicular spin torque oscillator (STO) could generate magnetization rotation at one end of the ferromagnetic strip that is also part of the STO. Our results demonstrate that the oscillation frequency of the spins along the wire maintains excellent fidelity while the spatial wavelength of the magnetic wave increases. The driving mechanism behind the propagation of the wave is found to be exchange-springs, which enables the propagation of the wave without the need for a '*carrier*' force, such as spin-transfer torque (STT) or spin Hall effect (SHE). Furthermore, we demonstrate that the gradient of the exchange energy drives the magnetic wave forward, while the in and out of plane anisotropy fields govern the shape of spin oscillation trajectories along the wire. Additionally, we show that stopping the oscillation at the STO end causes the wave to cease propagation after relaxation, and altering the STO rotational chirality leads to merging and annihilating domain walls of opposite winding numbers.

## Introduction

Much of the recent progress in spintronics involves transporting information and data storage via magnetization dynamics^1–4^. Most works on information transport utilize long-wavelength spin waves, which face obstacles such as short spin diffusion length and joule heating^5–9^ in metals where the magnetization precession cone angle is less than 10$^\circ$. To overcome these obstacles, there has been increasing interest in 360° spin precession in-plane (IP) with a cone angle of almost 90° through STT or SHE^10^. One of the feasible ways to produce such excitation is to use an STO. The free layer of an STO, IP magnetized due to shape or crystalline anisotropy, is extended beyond the source excitation in one direction to set up a nanowire geometry. The magnetization at the STO end undergoes a 360° IP rotation due to the STT effect from the perpendicular spin current, and the generated magnetization rotation in the strip propagates along the strip beyond the source region. While previous studies have addressed the concept of wave propagation by 360° IP rotation as the movement of homochiral domain walls in a dissipative or superfluid state^11–15^, our research aims to address crucial unanswered questions such as: what is the driving force behind this wave propagation? What happens when the source excitation is turned off, or its polarity is switched? Moreover, how does the wave behave under various conditions? Besides the macroscopic features of the wave, we also explained the interaction of IP and out-of-plane (OP) anisotropy fields which modifies the 3D trajectory of magnetic oscillation from one position to another in the NW when accounting for the demagnetizing field accurately.

## Results and discussions

**Figure 1:**
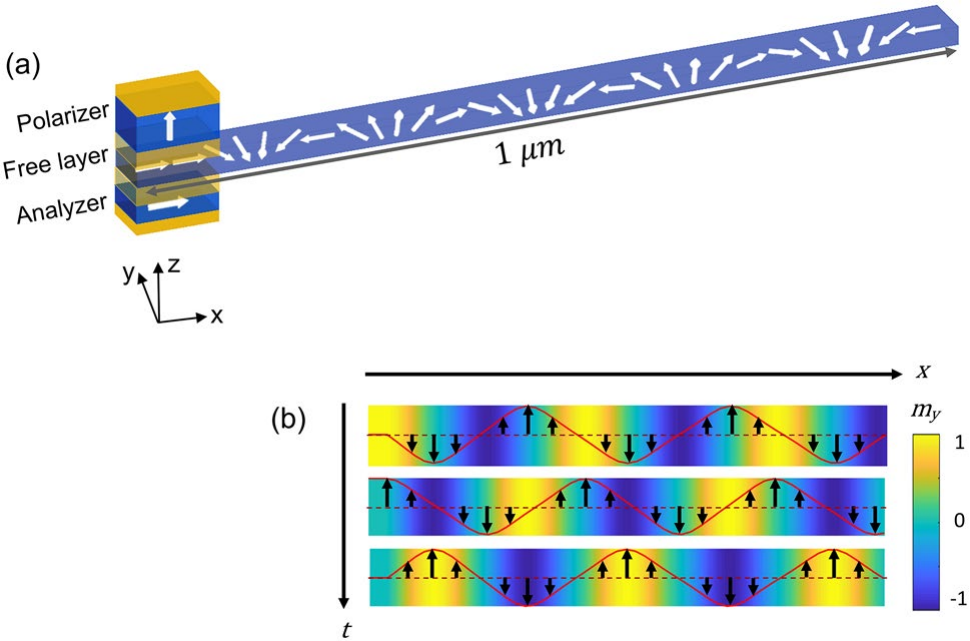
(**a**) Schematic of a perpendicular spin torque oscillator with an extended free layer. Magnetization wave propagates along the nanowire. NW length up to 1 µm is considered in this study. (**b**) Propagation through the part of the wire up to 250 nm is shown for half a time period in the ${m}_{y}$ component.

Consider a long strip of a thin magnetic film with magnetization confined inside the film plane by the planer anisotropy. Now, let us imagine a rotating magnetic field with constant angular velocity is locally applied to one end of the strip such that the magnetization of the strip would rotate along with it. This end is referred to as the source or STO end in the rest of the work since we propose using STO to achieve this rotation. Figure 1 shows the schematic of the STO with an extended free layer. When the magnetic moments at the STO end rotate IP due to the STT effect from the perpendicular spin filtered by the polarizer, the neighboring moments start rotation, too, as they experience a rotating exchange magnetic field. This process continues till the end of the wire. At the end of the strip, we assume sink/perfect absorption by considering a very high damping constant, so there is no reflection. One cycle of magnetization rotation at the STO end in time translates to a complete cycle or a 360° domain wall (DW) spatially. An increasing number of homochiral DWs pack in the wire with the continuous rotation of the source magnetization till the system reaches a steady state. Source magnetization oscillation frequency is set at 1 GHz for Fig. 1. Figure 1b shows the y-component of the magnetization orientation of the wire up to 250 nm with time, showing the wave propagation for half a period.

**Figure 2:**
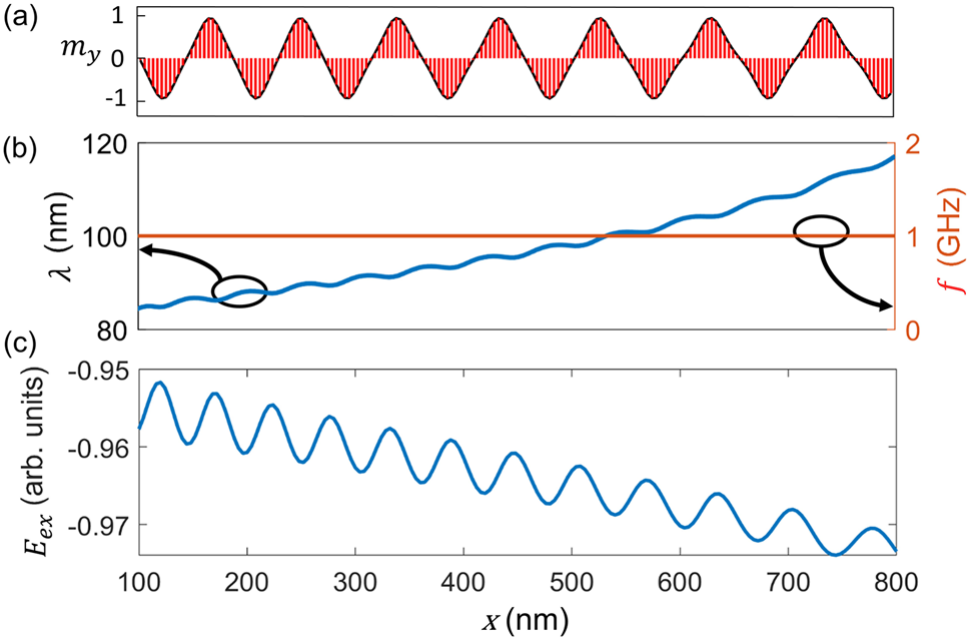
(**a**) ${m}_{y}$ component of the magnetization along the wire at the steady state. (**b**) At steady state, wavelength, $\lambda$ increases with distance away from the source (*x* = 0) while the oscillation frequency, $f$ remains constant. (**c**) Exchange energy, ${E}_{ex}$ goes down with distance.

Figure 2b shows that all spins in the NW oscillate at the source frequency in the steady-state. The explanation behind the equal frequency spatially in the steady-state comes from constraining the change in exchange energy, ${E}_{ex}$ with time. The Landau-Lifshitz-Gilbert (LLG) equation, the nonlinear equation, which is solved to calculate the magnetization dynamics, reaches a steady-state condition when $\frac{dE}{dt}=0$, where *E* is the total magnetic energy of the system. If all spins were not rotating at the same frequency at steady-state, neighboring moments would have increased phase difference among themselves, resulting in a rise of exchange energy which would have violated the steady-state condition. Reaching the steady-state condition does not entail the magnetic wave to stop propagating, however, as long as the source excitation remains active. In the steady-state condition, the magnetic energy at the STO end is higher than that of the other. The gradient of exchange energy forces the magnetic cycles to propel from the source to the other end (Fig. 2c). Alternatively, in other words, the wave propagates since the DWs closer to the source push the neighboring one down the descending energy slope. While ${E}_{ex}$ goes down, wavelength, $\lambda$ increases with distance (*x*) calculated from the source (*x* = 0), as seen in Fig. 2b. This increasing $\lambda$ trend is unsurprising since the decrease in ${E}_{ex}$ with distance necessitates the continuous decrease of phase difference among magnetic moments moving away from the source. The reduction of phase difference among spins in the transmission direction marks the trend of increasing wavelength with distance. Since the oscillation frequency of the moments is equal, a higher wavelength translates to a greater wave velocity towards the end. In the supplementary section, the video shows the wave propagation along the wire and corresponding precession frequency stabilized at the source frequency after a transient period. For a single frequency, there’s a range of wavelengths along the wire. Source frequency is varied and for each frequency, rotational wave with a different set of $\lambda$ appears and the $\lambda$ vs. frequency, $f$ is plotted in S1.

**Figure 3:**
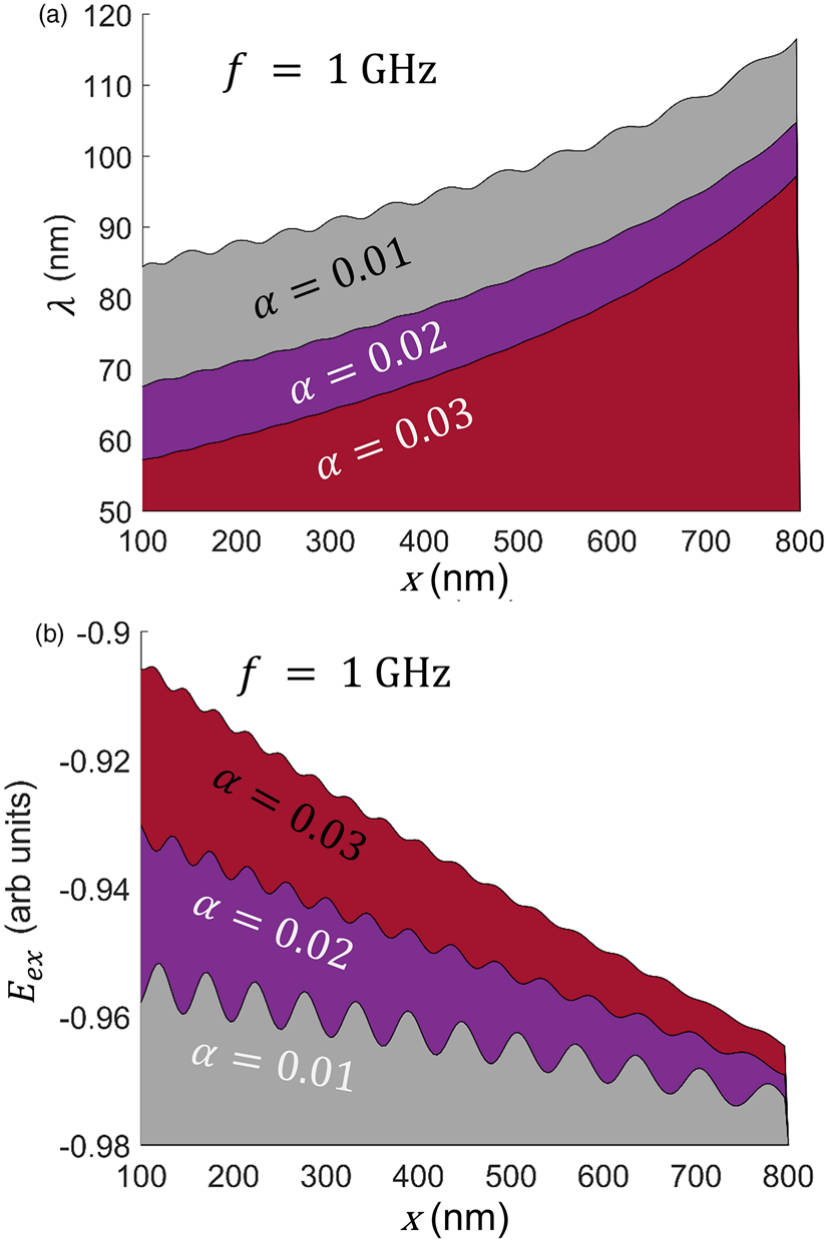
(**a**) Wavelength, $\lambda$ versus distance for various damping constants when the magnetization rotation frequency is 1 GHz. (**b**) Spatial profile of exchange energy, ${E}_{ex}$ vs. damping constant, $\alpha$ from 0.01 to 0.03. Higher $\alpha$ reduces the wavelength and increases the exchange energy.

From Fig. 3a, we found that $\lambda$ goes up when $\alpha$ goes down while keeping the same $\lambda -x$ trend of Fig. 2 intact. Smaller α reduces the rate of dissipating exchange energy between neighboring moments in a transient state, forcing a smaller phase angle between moments and resulting in a longer wavelength. The $\lambda -x$ relation shows an overlaying wavy pattern on top of the prominent underlying increasing trend of wavelength vs. distance. A similar wavy pattern is also strongly pronounced in ${E}_{ex}$ vs. *x* plot (Fig. 3b). Like wavelength, the magnitude of the overlapping pattern for ${E}_{ex}$ vs. *x* is much higher for lower $\alpha .$ This wavy nature results from the '*push and go*' movement of the DWs in the wire as the one near the source drives the adjacent one down the descending energy slope. As lower $\alpha$ results in spaced out wider DWs in the strip and higher $\alpha$ packs more DWs than that of smaller equivalent within the same length, DWs' wavy movement gets tightened with increasing damping constant, and therefore, less pronounced wavy pattern in a highly damped wire.

**Figure 4:**
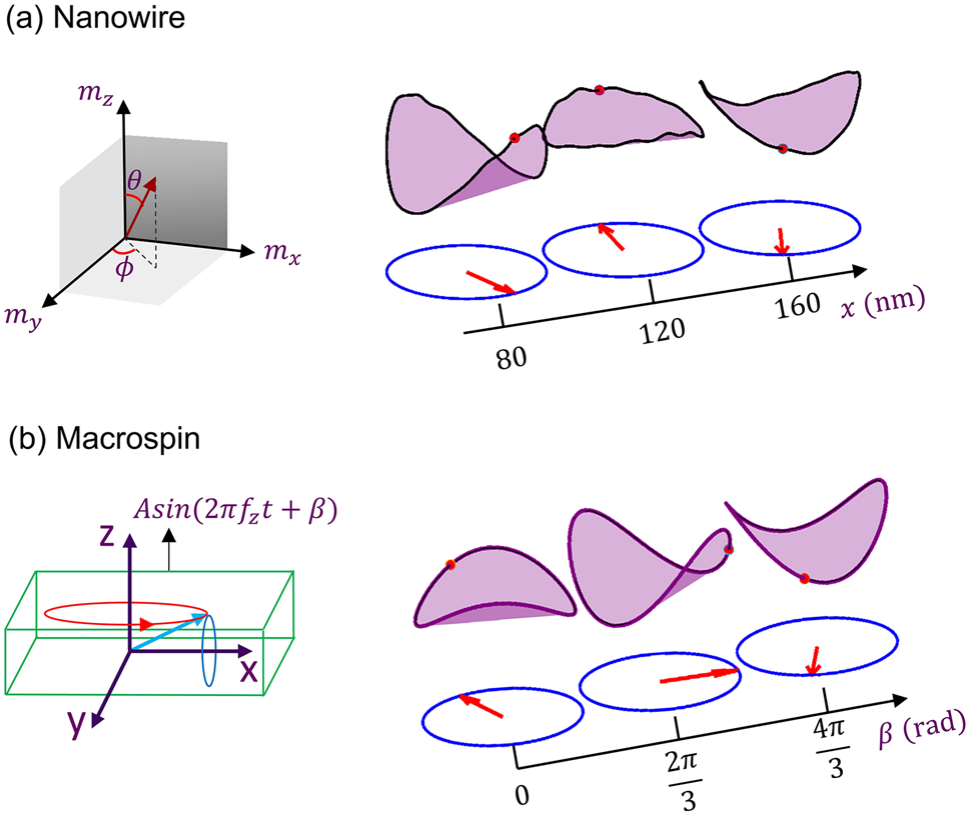
(**a**) 3D oscillation trajectory changes along the nanowire from one position to another. (**b**) Change in spatial oscillation trajectory in the NW is explained using a modified macrospin model of spin torque oscillator. A perpendicular sinusoidal field is applied to the free layer of STO. Changing the phase angle of the applied field mimics the changes in the nanowire.

The propagating magnetizing rotation along the strip is effectively exchange springs, enabling the propagation of the wave without any '*carrier force*' such as STT or SHE. Although exchange-energy derived force causes the propagation, other energies are crucial to understanding this magnetic wave propagation. When a spin precesses, it will have a tiny OP component bringing the hard OP anisotropy into play which is very strong due to thin wire geometry. Moreover, due to the NW geometry, magnetic moments prefer pointing along the elongated, i.e., transmission direction. These IP and OP anisotropy fields effect the overall magnetodynamics of the wave. In the NW, we found that the one-period oscillation trajectory changes from hyperparabolic paraboloid at one location to almost circular at another point and to the parabolic shape later (Fig. 4a). We try to unravel the rationale of varying trajectories through IP and OP precessions in the NW with a modified macrospin model of an STO. In the macrospin STO model, assuming only hard OP anisotropy of the ferromagnet results in a perfect circular oscillation trajectory with a fixed value of the OP component, ${m}_{z},$ the z-component of the magnetic moment^16,17^. However, including an IP anisotropy, a parabolic trajectory emerges with OP oscillation frequency doubling that of IP components. With the increase of IP anisotropy, the trajectory becomes more parabolic, but it does not change the shape^18^. In the NW, OP oscillation frequency is also double that of IP; however, its trajectories vary across the locations. To understand what happens in the NW, we incorporate changes in the macrospin model (Fig. 4b). We fix the anisotropies in the macro-model and add a sinusoidal OP field of the form: $H_{z} = A\sin \left( {2\pi f_{z} t + \beta } \right)$. The magnitude of *A* is very low compared to the anisotropy values. Frequency, ${f}_{z}$ is twice the source frequency. With the change in the phase term, $\beta ,$ changes in the trajectory resembles that of various positions in the wire. Adding this phase in macrospin model stems from the perception that the exchange field in the OP direction for NW would have a phase delay between the neighboring moments. To validate our assumption, we also checked the OP field of two nearby spins of the NW and found out the phase delay between them.

**Figure 5:**
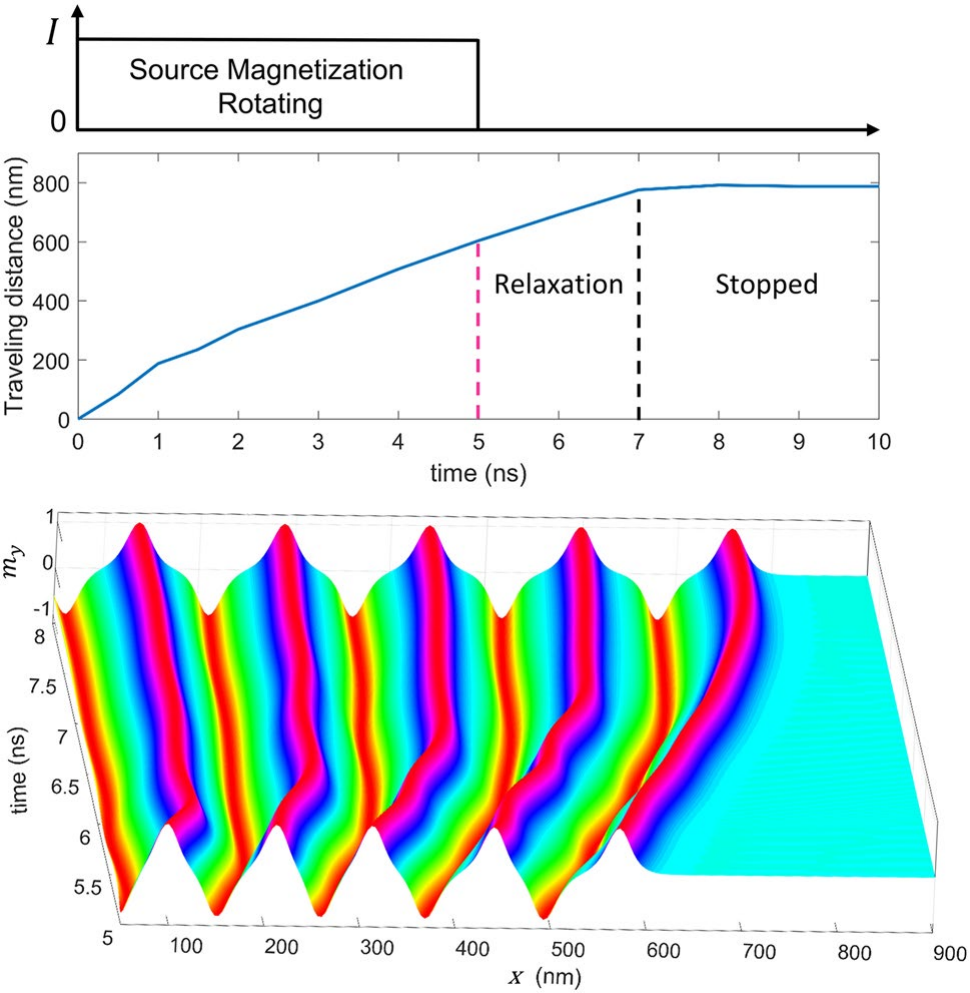
Magnetization wave relaxes to stable minimum energy configuration within 2 ns of the stopping of source magnetization rotation.

Now, we analyze what happens when we stop the source excitation or change the chirality of source rotation. In the first case, the magnetic moments at the source end were let to oscillate at 1 GHz for the first five periods, and in Fig. 5, we see the five spatial cycles as a result. After stopping the excitation, the magnetic moments relax into a stable local minimum energy configuration with the DWs further separating from each other. Initially, the wavelengths close to the source were shorter than those away to facilitate wave propagation. After relaxation within around 2 ns, there is no further movement of the cycles, and the width of the DWs becomes uniform, suggesting a zero gradient in exchange energy vs. distance. This static state shows the importance of continuous excitation to maintain magnetic wave propagation. Restarting the source-magnetization rotation will resume the magnetic wave propagation.

**Figure 6:**
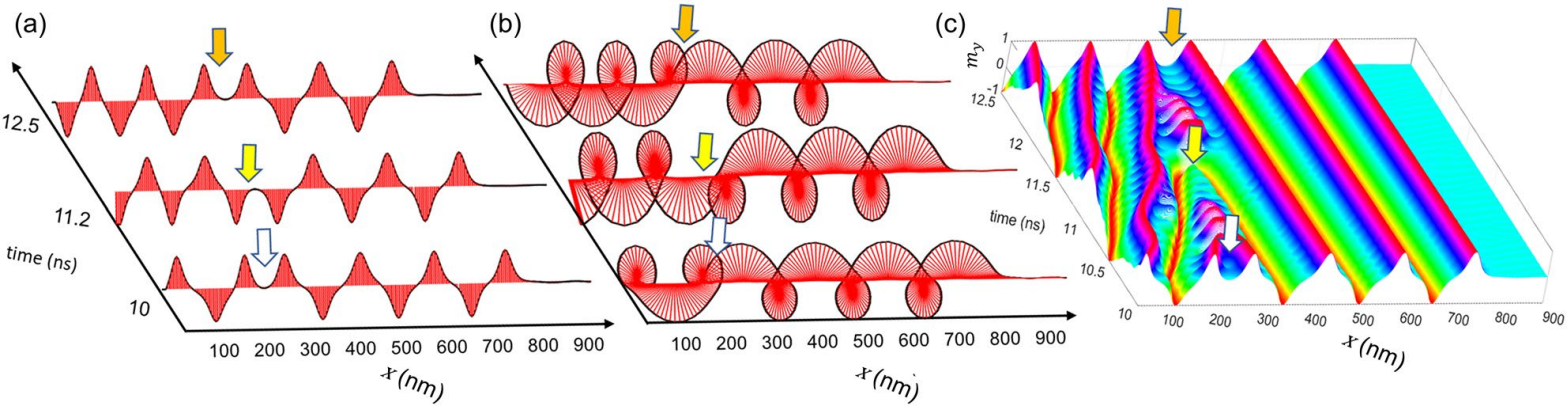
Oppositely wounded domain walls merge and annihilate each other after the source magnetization changes its rotation chirality. The ${m}_{y}$ component is represented in (**a**) wave (**b**) helix format for the three-time stamps when the merging begins. (**c**) Plots the component for intermediate time stamps, too, showing the generation of spin waves during the annihilation process.

For the second case, the source-magnetization changes rotation chirality (clockwise (CW) to anticlockwise (CCW)) after a few ns. The opposite chirality of the source excitation makes new DWs, which collide with the old DWs having the opposite winding number to the new ones and annihilate them (Fig. 6)^19^. This collision and annihilation processes continue so long as all DWs with old winding get '*swallowed'* by newer ones. In the three subplots of Fig. 6, the arrows indicate the intersection of old and new cycles. Figure 6a plots the y-component of the magnetization, ${m}_{y}$ for three-time stamps. First, two positive half-cycles come closer, merge, and annihilate, then the next two negative half-cycles merge, and later two positive half cycles combine. The change in wave polarization is more apparent in the helix representation of IP magnetization in Fig. 6b. Figure 6c plots the ${m}_{y}$ component for the intermediate time stamps. Figure 6c shows that whenever two cycles of opposite winding numbers merge, a spin wave (SW) is generated, which oscillates around the intersection^19^. SW, which is generated due to the merge, passes through the wire, and decays its magnitude with time in the damped wire. The SW also perturbs the unaffected DWs as it passes through them, even though the movement of the DW from their mean position is small. The overall stability of the unaffected DWs is minimally disturbed due to this SW propagation. So, the CW cycles which have not collided yet have not moved. Therefore, the traveling distance of the magnetization wave remains constant if there is continuous swapping of the source excitation chirality at regular intervals.

## Conclusion

A rotating magnetic wave propagates through a nanowire by driving a complete 360° in-plane oscillation at one end of the ferromagnetic wire. This ferromagnetic strip could be the free layer of a perpendicular STO extending beyond the oscillator region. STO sets up a rotation at one end of the wire, which results in the generation and propagation of the magnetic wave. The wave is characterized by constant frequency throughout and an increasing wavelength in the transmission direction, causing a gradient in the exchange energy. The force from the exchange energy pushes the wave forward. Though the wave propagation is driven by exchange-energy gradient, the interplay of exchange and demagnetizing field alters the oscillation trajectories of the moments through the strip. After stopping the source magnetization oscillation, wave propagation ceases, while after switching the source rotational chirality, merging and annihilation of magnetic cycles of opposite winding numbers occurs. For a conventional linear spin wave, for example, 1D chain of moments exhibiting spin-wave behavior, the precession of the moments is position dependent, and this non-uniform precession creates wave-like solutions. However, for this study, the linearization of the LLG equation to generate dispersion relation like that of conventional wave is not possible since the equation is inherently non-linear, the oscillation magnitude is full scale IP and the precession frequency is not position dependent at steady state, rather determined by source frequency due to exchange force. In terms of the applicability of this rotational magnetic wave, our thorough theoretical investigation will be useful to uniquely modulate the magnetic wave for practical purposes. One possible way to use the source frequency as a state variable to transmit data through the wire. Information can be passed on from one side of the wire to another without the charge current flowing through it. Studies like this will add further interest to the promising area of spintronics, which would pave the way for further novel theories and applications.

## Simulation method

$1 \; \upmu m\times 10 \; \text{nm}\times 2 \; \text{nm}$ Free layer of the STO is discretized using the grid size of $4\; \text{nm}\times 2 \; \text{nm}\times 2 \; \text{nm}$. Micromagnetic simulation has been done to calculate the magnetization dynamics of the ferromagnetic strip by solving the following LLG equation (including the precessional and damping torque terms) for the spin in each cell:

$$\frac{d{\varvec{m}}}{dt}= -\gamma \left({\varvec{m}}\times {{\varvec{H}}}_{{\varvec{e}}{\varvec{f}}{\varvec{f}}}\right)+\alpha {\varvec{m}}\times \frac{d{\varvec{m}}}{dt}$$

***m*** = $\frac{{\varvec{M}}}{{M}_{s}}$ is the normalized magnetic moment for each grid point. ${M}_{s}$ is the saturation magnetization of the ferromagnet set at 800 emu/cc. $\gamma$ is the gyromagnetic ratio for electron. $\alpha$ is the damping constant and is set to $0.01$ unless stated otherwise. However, to eliminate the reflection of the magnetic wave, an absorbing boundary condition (BC) has been assumed for the other end of the wire, which is not coupled to the STO. For the last few cells, a very high value of $\alpha$ = 0.5 is considered to replicate the absorbing BC so that there is no reflection. ${{\varvec{H}}}_{{\varvec{e}}{\varvec{f}}{\varvec{f}}}$ is the effective magnetic field, which includes the exchange field and demagnetizing field. The exchange field is defined as the ferromagnetic interaction field between two neighboring spins and is given by ${{\varvec{H}}}_{{\varvec{a}}}=\frac{2A}{{M}_{s}^{2}}({\nabla }^{2}{\varvec{m}})$, where A is the exchange energy density constant set at $1.6 \mu \frac{erg}{cm}$. The demagnetizing field for each cell is calculated by the dot product of the effective demagnetizing tensor and the corresponding magnetization. Effective demagnetizing tensor is computed using the equations in^20^. A fast Fourier transform (FFT) is adopted to reduce the computational burden from $O({N}^{2})$ to $O(NlogN)$^21^. In this work, we manually rotate the free layer magnetization at the STO end to mimic the STO effect. Since this work is focused on analyzing the wave resulting from full-scale IP magnetization rotation at one end, we have excluded the perpendicular STO simulation to reduce the computational complexity and to consolidate our research into the properties of the wave. We have assumed the moments at the source end to follow an ideal IP propagation, such as ${m}_{x}=cos\left(2\pi ft\right), {m}_{y}=\mathrm{sin}\left(2\pi ft\right)$ while zero OP component is considered. $f$ is the source frequency. This '*ideal'* source spin exchange forces the neighboring moments to follow it as long as the frequency, $f$ is not very high to allow the adjacent moments to synchronize with the source. We have assumed the driving frequency such that the neighboring moments could follow the source spin while using typical material properties. Increasing source frequency increases the phase angle separation between the neighboring moments, result in reduction of wavelength as seen in Fig. S1 (supplementary section). In the case of a very high source frequency, the moments will no longer be able to follow the source spin, synchronization will be broken, and '*chaotic*' spin waves will result, which is not studied in this work. The maximum allowable frequency for the synchronization depends on the material properties as it is inherently tied to the exchange length in magnetics. Even though the '*ideal*' source spin is assumed to have zero OP component, the other moments in the wire follow the LLG equation, and they develop a small ${m}_{z}$ component to precess and the resulting complex magnetodynamics is explained in detail before (Fig. 4). If STO also drove the source spin, it would also have generated a tiny OP component, but it would not change the findings of this paper. Changing the applied current density would result in different driving frequencies. The procedure to calculate the wavelength ($\lambda )$ vs. position in Figs. 2 and 3 is as follows. For each simulation time step, the spatial difference between the two peak points of ${m}_{y}$ component (magnetization with the same phase angle) is calculated as the wavelength for the spins between those peak points for that time stamp. One 360° DW is nucleated at the source in one temporal cycle, and another is completely absorbed at the other end. The average of spatial wavelengths in one time period is taken as steady state wavelength in $\lambda$ versus position relation. While the source frequency, $f$ is taken as input, the frequency of other spins are calculated using the formulae $f=\frac{1}{2\pi } (\frac{d\phi }{dt})$ where $\phi$ is the IP phase angle.

### Supplementary Information

Supplementary Information.

## Data Availability

The datasets generated and analyzed during the current study are available from the corresponding author on reasonable request.
