# Supplementary material for: Micromagnetic insights on in-plane magnetization rotation and propagation of magnetization waves in nanowires
Source: Sci Rep. 2023 Aug 18;13:13438. doi: 10.1038/s41598-023-40515-9 (PMC10439149; doi:10.1038/s41598-023-40515-9)
Supplement: Supplementary file 1 — Supplementary Information. [file 41598_2023_40515_MOESM1_ESM.pptx]

## Slide 1
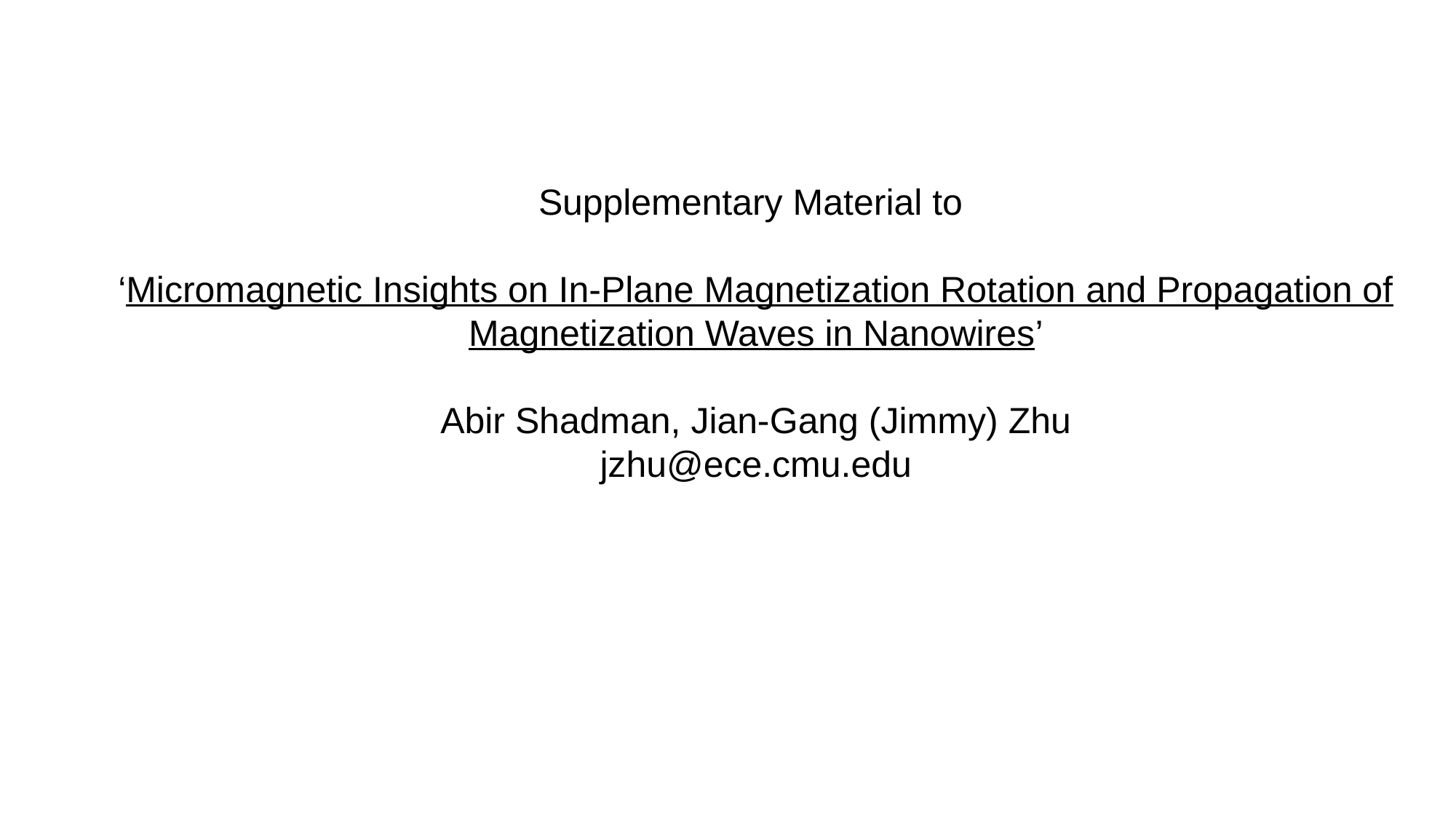

Supplementary Material to
‘Micromagnetic Insights on In-Plane Magnetization Rotation and Propagation of Magnetization Waves in Nanowires’
Abir Shadman, Jian-Gang (Jimmy) Zhu
jzhu@ece.cmu.edu

## Slide 2
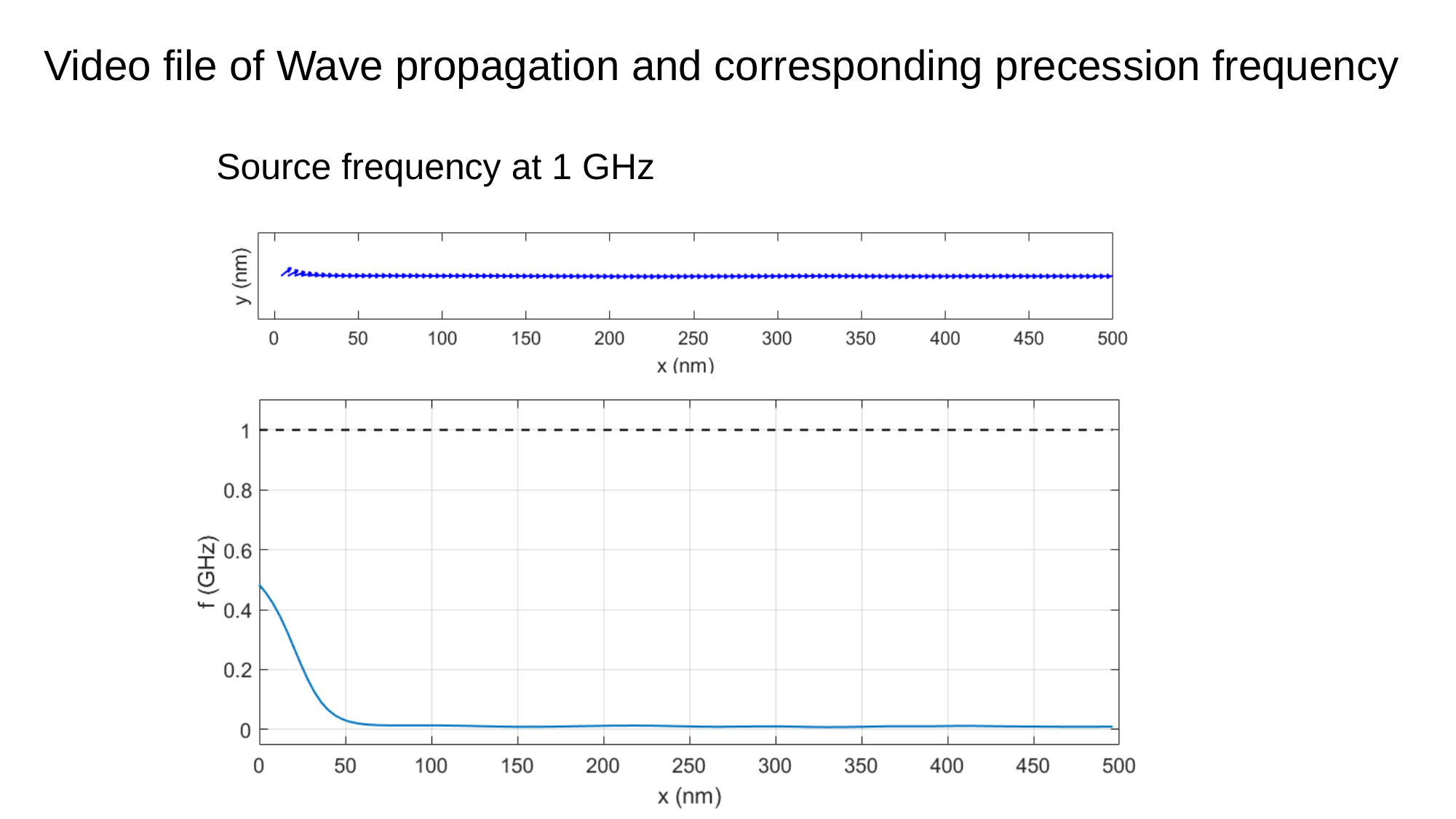

# Video file of Wave propagation and corresponding precession frequency
Source frequency at 1 GHz

## Slide 3
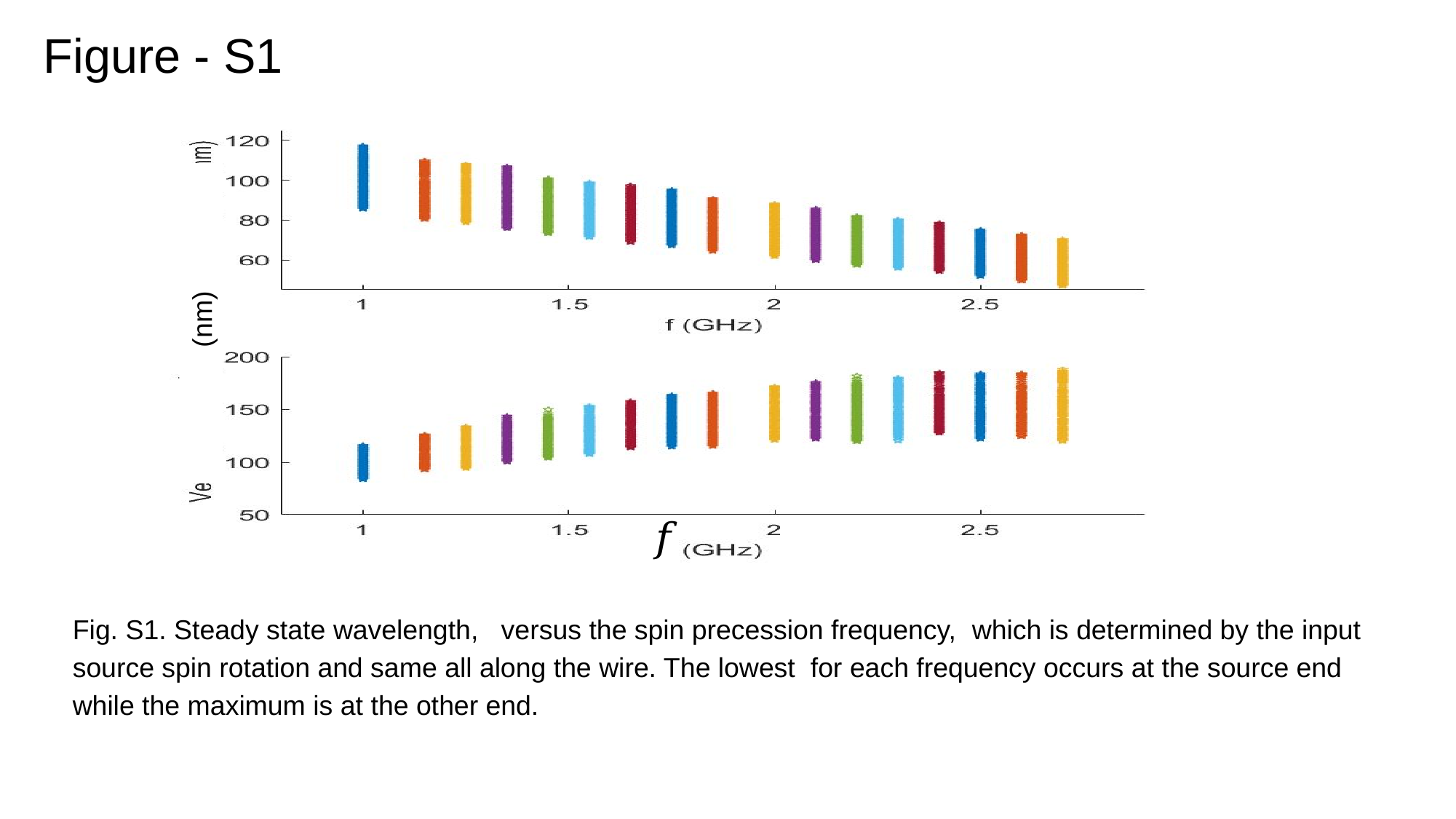

Figure - S1
